# Treatment efficacy, treatment failures and selection of macrolide resistance in patients with high load of *Mycoplasma genitalium* during treatment of male urethritis with josamycin

**DOI:** 10.1186/s12879-015-0781-7

**Published:** 2015-02-03

**Authors:** Alexander Guschin, Pavel Ryzhikh, Tatiana Rumyantseva, Mikhail Gomberg, Magnus Unemo

**Affiliations:** Department of Molecular Diagnostics and Epidemiology, Central Research Institute for Epidemiology, Moscow, Russia; Moscow Scientific and Practical Center for Dermatovenerology and Cosmetology, Moscow, Russia; WHO Collaborating Centre for Gonorrhoea and other STIs, National Reference Laboratory for Pathogenic Neisseria, Örebro University Hospital, Örebro, Sweden; Department of Laboratory Medicine, Microbiology, School of Medicine, Örebro University, Örebro, Sweden

**Keywords:** *Mycoplasma genitalium*, Treatment, Treatment efficacy, Treatment failure, Antimicrobial resistance, 23S rRNA, Josamycin, Macrolides, Russia, Male urethritis

## Abstract

**Background:**

Azithromycin has been widely used for *Mycoplasma genitalium* treatment internationally. However, the eradication efficacy has substantially declined recent decade*.* In Russia, josamycin (another macrolide) is the recommended first-line treatment for *M. genitalium* infections, however, no data regarding treatment efficacy with josamycin and resistance in *M. genitalium* infections have been internationally published. We examined the *M. genitalium* prevalence in males attending an STI clinic in Moscow, Russia from December 2006 to January 2008, investigated treatment efficacy with josamycin in male urethritis, and monitored the *M. genitalium* DNA eradication dynamics and selection of macrolide resistance in *M. genitalium* during this treatment.

**Methods:**

Microscopy and real-time PCRs were used to diagnose urethritis and non-viral STIs, respectively, in males (n = 320). *M. genitalium* positive patients were treated with recommended josamycin regimen and treatment efficacy was monitored using quantitative real-time PCR. Macrolide resistance mutations were identified using sequencing of the 23S rRNA gene.

**Results:**

Forty-seven (14.7%) males were positive for *M. genitalium* only and most (85.1%) of these had symptoms and signs of urethritis. Forty-six (97.9%) males agreed to participate in the treatment efficacy monitoring. All the pre-treatment *M. genitalium* specimens had wild-type 23S rRNA. The elimination of *M. genitalium* DNA was substantially faster in patients with lower pre-treatment *M. genitalium* load, and the total eradication rate was 43/46 (93.5%). Of the six patients with high pre-treatment *M. genitalium* load, three (50%) remained positive post-treatment and these positive specimens contained macrolide resistance mutations in the 23S rRNA gene, i.e., A2059G (n = 2) and A2062G (n = 1).

**Conclusions:**

*M. genitalium* was a frequent cause of male urethritis in Moscow, Russia. The pre-treatment *M. genitalium* load might be an effective predictor of eradication efficacy with macrolides (and possibly additional antimicrobials) and selection of macrolide resistance. Additional *in vivo* and *in vitro* data are crucial to support the recommendation of using josamycin as first-line treatment for *M. genitalium* infections in Russia. It would be valuable to develop international *M. genitalium* management guidelines, and quantitative diagnostic PCRs determining also *M. genitalium* load and resistance mutations (for macrolides and ideally also moxifloxacin) should ideally be recommended.

## Background

*Mycoplasma genitalium* has in recent decades been shown to be a frequent cause of urethritis in men and urethritis, cervicitis, endometritis, pelvic inflammatory disease, and possibly infertility in women. In patients with non-gonococcal urethritis (NGU), *M. genitalium* is commonly the second most frequent etiology, i.e., after *Chlamydia trachomatis* [1-6]. In the general population, the prevalence of *M. genitalium* ranges from approximately 1% to 3%, and among men with symptomatic non-chlamydial, non-gonococcal urethritis (NCNGU), the prevalence ranges from 15% to 35% [1,7,8].

Still, no international management guidelines for *M. genitalium* infections have been published. The recommended treatments of male NGU in the European guideline include tetracyclines and macrolides [9]. However, despite being effective *in vitro* tetracyclines have shown a low eradication rate for *M. genitalium* clinically [1,10-13]. Azithromycin 1 g single oral dose has been widely used. However, despite that large, appropriate randomized clinical trials are lacking an extended azithromycin regimen has been indicated to be slightly more effective or at least equally effective in eradicating *M. genitalium*, i.e., azithromycin 500 mg on day 1 followed by 250 mg daily on days 2–5 [1,11,13-15]. Worryingly, the eradication rate of *M. genitalium* after azithromycin 1 g therapy has substantially declined during the last decade [1,10,12-14,16]. Recently, it was also reported that azithromycin 1 g therapy was associated with selection of azithromycin resistance in *M. genitalium* [14]*.* It was also most recently hypothesized that treatment failure can be associated with a higher load of *M. genitalium* in the clinical specimens [17]. *M. genitalium* strains resistant to azithromycin contain resistance mutations in region V of 23S rRNA, which is encoded be a single copy gene in *M. genitalium* [1,10,13,14,18]. Moxifloxacin is the most commonly used second-line regimen in patients failing azithromycin therapy. However, the side-effects, cost, and the risk of selection of resistance limit the moxifloxacin use [1,13,15,19,20].

In Russia, the 16-membered lactone ring macrolide josamycin (500 mg three times daily, 10 days) is the nationally recommended first-line treatment for *M. genitalium* infections and it is widely used mainly in the treatment of NGU, *C. trachomatis* infections and *M. genitalium* infections, including in pregnancy [21]. Josamycin is also included in the second-line treatment in the European *C. trachomatis* management guideline [22], however, in general josamycin is very rarely used in the majority of European countries. Despite that the *in vitro* susceptibility to josamycin in *M. hominis* and ureaplasmas are high [23,24], very limited *in vitro* data regarding josamycin susceptibility in *M. genitalium* have been published [25], which have not indicated a higher activity than azithromycin. Furthermore, no data regarding treatment efficacy of *M. genitalium* infections with josamycin have been published in the international (English language) literature. Moxifloxacin 400 mg daily, 10 days can be used as a second-line regimen for treatment of *M. genitalium* infections in Russia.

The aims of this study were to examine the prevalence of *M. genitalium* in males attending an STI clinic in Moscow, Russia from December 2006 to January 2008, investigate the treatment efficacy with josamycin in male urethritis, and to monitor the *M. genitalium* DNA eradication dynamics and selection of macrolide resistance in *M. genitalium* during treatment of male urethritis with josamycin.

## Methods

The work was performed at the Department of Molecular Diagnostics and Epidemiology, Central Research Institute for Epidemiology, Moscow, Russia.

### Study population

Consecutive males (n = 320) attending one of the venereologist at a sexually transmitted infection (STI) clinic in Moscow, Russia, from December 2006 to January 2008, were enrolled in the study. The males attended the STI clinic due to urethral symptoms or having had unprotected sexual intercourse with new or multiple contact(s). The mean age of the males was 31 years (median age: 30 years; range: 18 to 66 years). All males signed an informed consent form, the males were clinically examined and two urethral swabs were sampled from each patient, i.e. for microscopy and PCR, respectively.

### Ethical approval

Ethical approval for the study was obtained by the Central Research Institute for Epidemiology, Moscow, Russia.

### Laboratory diagnostics (microscopy and PCR)

Microscopy (1000× magnification) of Gram-stained smears was performed. The presence of ≥5 polymorphonuclear leucocytes (PMNL) per high power field (hpf), in at least 5 hpf, was interpreted as urethritis. For diagnosis, *M. genitalium* DNA was detected by the real-time PCR assay AmpliSens® *M.genitalium*-FRT, and the AmpliSens® *N.gonorrhoeae*-FRT, AmpliSens® *C.trachomatis*-FRT, and AmpliSens® *T.vaginalis*-FRT assays (InterLabServices Ltd, Moscow, Russia) were used to diagnose other non-viral STIs. The DNA isolation and PCR assays, which have been previously evaluated, were performed in accordance with the instructions from the manufacturer (InterLabServices Ltd, Moscow, Russia) and as previously described [26-29].

### Antimicrobial treatment of patients

In accordance with the Russian treatment guidelines, all *M. genitalium* positive patients were treated with josamycin 500 mg three times daily, 10 days [21]. Treatment failures with the josamycin regimen were administered moxifloxacin 400 mg daily, 10 days. Patients positive for any other STI (according to the laboratory test results) were also treated in accordance with the Russian treatment guidelines [21].

### Monitoring of josamycin treatment efficacy

All *M. genitalium* positive patients that did not have any other STI, any complicated urogenital infection, or had been administered any antimicrobials in four weeks prior to enrollment, were invited for monitoring of treatment efficacy. In participating patients, one urethral swab (for microscopy) and one first-void urine specimen (for PCR) were sampled, in a standardized and quality assured manner, on treatment onset, 3rd and 8th day of treatment, and 2nd and 14th days after completed treatment. The first-void urine was collected in 50 mL sterile disposable plastic containers, with a calibration scale in mL on the wall of the container. According to instructions and using these containers, the patients collected 15–20 mL of first-void urine (predominantly morning urine and the patients should not had urinated 3 h before). Valid data regarding the time since last micturition was unfortunately not possible to obtain. At each visit, compliance of the therapy was ensured. To quantify the *M. genitalium* load in these clinical specimens, three in house prepared DNA calibrators based on the *gyrB* gene (10^2^, 10^3^, 10^4^ genome equivalents (geq)/reaction) were included in the real-time PCR assay AmpliSens® *M.genitalium*-FRT (InterLabService Ltd, Moscow, Russia) [26]. The *M. genitalium* concentration detected was expressed as number of geq [log_10_] per 1 mL of transport media and the *M. genitalium* load in each specimen was arbitrarily categorized as low (≤4 geq/mL [log_10_]), moderate (>4 - <6 geq/mL [log_10_]) or high (≥6 geq/mL [log_10_]). The *M. genitalium* positive samples were subsequently preserved at −70°C, that is, prior to sequencing for identification of macrolide resistance mutations.

### Identification of macrolide resistance mutations

For identification of macrolide resistance mutations, domain V of the 23S rRNA gene in *M. genitalium* was sequenced using the previously described primers Mg23S-1992F and Mg23S-2138R [18].

## Results

### Symptoms/signs, microscopy results and STI etiological agents in all males (n = 320)

Among the 320 males, 222 (69.4%) had symptoms/signs and microscopically confirmed urethritis, and 49 (15.3%) had no symptoms/signs but microscopically verified urethritis (Table 1). Accordingly, in total 271 cases of urethritis were confirmed. *C. trachomatis*, *M. genitalium*, *Neisseria gonorrhoeae* and *Trichomonas vaginalis* was detected in 102 (37.6%), 46 (17.0%), 29 (10.7%) and 9 (3.3%), respectively, of the 271 urethritis cases. *C. trachomatis* and *M. genitalium* was also detected in eight and five, respectively, of the males lacking symptoms/signs and having ≤5 PMNLs/hpf.

**Table 1:** **Symptoms/signs, polymorphonuclear leucocytes (PMNLs), and detected STI etiological agents in males (n = 320) attending an STI clinic in Moscow, Russia, from December 2006 to January 2008**

**Clinical characteristics**	**Number of patients**	***Chlamydia trachomatis*** **positive**	***Mycoplasma genitalium*** **positive** ^**a**^	***Neisseria gonorrhoeae*** **positive**	***Trichomonas vaginalis*** **positive**
**Symptoms/signs and**	222	75	40	27	6
**PMNLs ≥5**					
**Symptoms/signs and**	0	0	0	0	0
**PMNLs <5**					
**No symptoms/signs and**	49	27	6	2	3
**PMNLs ≥5**					
**No symptoms/signs and**	49	8	5	0	0
**PMNLs <5**					
Total	320	110	51	29	9

^a^Four patients positive for *M. genitalium* as well as some additional STI etiological agent, i.e. *C. trachomatis* (n = 2), *T. vaginalis* (n = 1), and Herpes simplex virus type 2 (n = 1), were excluded.

### *Mycoplasma genitalium* positive males

In total, *M. genitalium* was detected in 51 (15.9%) of the 320 includes males. However, four of the *M. genitalium* positive males were also positive for some additional STI etiological agent, that is, *C. trachomatis* (n = 2), *T. vaginalis* (n = 1), and Herpes simplex virus type 2 (n = 1). For the treatment monitoring, these four males were excluded. However, no males were excluded due to having a complicated urogenital infection or taking antimicrobials during the four weeks prior to enrollment. Accordingly, 47 (14.7%) of the 320 males were positive for *M. genitalium* only, which corresponded to 44 (16.2%) of the confirmed 271 urethritis cases. Of these 47 *M. genitalium* positive patients, 40 (85.1%) had symptoms of urethritis and ≥5 PMNL/hpf, four (8.5%) were asymptomatic but had ≥5 PMNL/hpf, and three (6.4%) had no symptoms and <5 PMNL/hpf. Twenty-two (46.8%) and 15 (31.9%) of the 47 patients had ≥10 and >50 PMNL/hpf, respectively.

### Monitoring of treatment efficacy with josamycin

Forty-six (97.9%) of the 47 males positive for only *M. genitalium* agreed to participate and attended most of the visits for the monitoring of treatment efficacy with josamycin. The mean age of these participants was 30 years (median age: 30 years; range: 18 to 41 years). The results of the treatment monitoring are summarized in Table 2.

**Table 2:** **Eradication of** ***Mycoplasma genitalium*** **DNA during treatment with josamycin in 46 males with urethritis**

**Bacterial load pre-treatment**	***M. genitalium*** **DNA concentration (geq/mL [log**_**10**_ **])**	**No. (%) of** ***M. genitalium*** **positive patients**				
		**0 d/t**	**3 d/t**	**8 d/t**	**2 d/at**	**10-14 d/at**
Low	≤4	10 (100)	3 (30)	0 (0)	0 (0)	0 (0)
Moderate	>4 - <6	29 (100)	15 (51.7)^b^	3 (10.3)	2 (6.9)^d^	0 (0)
High	≥6	6 (100)	5 (83.3)	2 (33.3)^c^	3 (50)	3 (50)
Total no. of patients examined		45^a^	45	45	45	14^e^

geq/mL (log_10_), log_10_ of genome equivalents/mL; d/t, days of treatment; d/at, days after completed treatment.

^*a*^Sample from one patient was not available. At 3 d/t, this patient had 3.1 geq/mL [log_10_], and at the three subsequent visits he was *M. genitalium* negative.

^*b*^Sample from one patient was not available. This patient was asymptomatic, *M. genitalium* negative but demonstrated microscopic signs of urethritis on the three subsequent visits.

^*c*^Sample from one patient was not available. This was treatment failure A, see Table 3.

^*d*^Sample from one patient was not available. The patient reported to be asymptomatic on the phone and at 3 d/t and 8 d/t, no symptoms, signs or *M. genitalium* DNA was detected.

^*e*^Thirty-two patients did not attend. However, all of the non-attending patients were *M. genitalium* negative already at earlier visits (both at 8 d/t and 2 d/at) and also reported to be completely asymptomatic on phone.

Briefly, 10 (22.2%), 29 (64.4%) and six (13.3%) of the patients had a low (≤4 geq/mL [log_10_]), moderate (>4 - <6 geq/mL [log_10_]) and high (≥6 geq/mL [log_10_]), respectively, pre-treatment *M. genitalium* load. All the available pre-treatment *M. genitalium* specimens (n = 45) had a wild-type 23S rRNA gene sequence. After only three days of treatment, 48.9% (22/45) of the examined patients were *M. genitalium* negative, and after eight days of treatment 88.9% (40/45) were negative. The elimination of *M. genitalium* DNA was substantially faster in patients with lower pre-treatment *M. genitalium* load. For example, all the 13 (100%) patients with low pre-treatment bacterial load were *M. genitalium* negative already after three days of treatment, and only 6.9% (2/29) of patients with moderate pre-treatment *M. genitalium* load had detectable levels of *M. genitalium* DNA two days post-treatment, which was eliminated 10–14 days post-treatment. However, of the six patients with high pre-treatment *M. genitalium* load three (50%) remained positive 10–14 days after completed treatment (Table 2). All these three patients repeatedly assured full compliance to treatment and not having had any sexual contacts between initiation of josamycin therapy and follow up visit 10–14 days after completed treatment. Accordingly, the eradication rate with the josamycin treatment was 43/46 (93.5%). Details regarding the three treatment failures are provided in Table 3.

**Table 3:** **Details of three failures to treat** ***Mycoplasma genitalium*** **urethritis with josamycin, including bacterial load in log**_**10**_ **genome equivalents/mL (23S rRNA gene), symptoms and microscopy results at different visits**

**Patients**	**Sexual orientation**	**0 d/t**	**3 d/t**	**8 d/t**	**2 d/at**	**10-14 d/at**
Case A^a^	Heterosexual	6.4 (WT)	4.7 (WT)	NA	2.1 (A2059G)	3.2 (A2059G)
		Severe urethritis symptoms, 25–30 PMNL/hpf	Less severe urethritis symptoms, 10–15 PMNL/hpf	Mild itching only, 2–3 PMNL/hpf	Asymptomatic, <5 PMNL/hpf	Asymptomatic, <5 PMNL/hpf
Case B^a^	Heterosexual	6.2 (WT)	2.5 (WT)	0.8 (NA)	2.0 (A2062G)	1.9 (A2062G)
		Severe urethritis symptoms, 60 PMNL/hpf	Less severe urethritis symptoms, 20–30 PMNL/hpf	Mild discharge and itching, 15–20 PMNL/hpf	Asymptomatic, 10–12 PMNL/hpf	Severe urethritis symptoms, 100 PMNL/hpf
Case C^a^	Heterosexual	7.2 (WT)	7.0 (WT)	4.3 (WT)	5.0 (A2059G)	7.5 (A2059G)
		Asymptomatic, <5 PMNL/hpf	Asymptomatic, <5 PMNL/hpf	Asymptomatic, <5 PMNL/hpf	Asymptomatic, <5 PMNL/hpf	Severe urethritis symptoms, 40–60 PMNL/hpf

d/t, days of treatment; d/at, days after completed therapy; WT, wild-type 23S rRNA gene sequence; NA, not assessed (specimen not available and too low concentration of *M. genitalium* DNA for 23S rRNA gene sequencing, respectively); PMNL/hpf, polymorphonuclear leucocytes/high power field.

^*a*^All three patients repeatedly reassured that they had not had any unprotected sexual contacts between initiation of the josamycin treatment and test of cure. All patients were subsequently successfully treated with moxifloxacin 400 mg single oral dose daily in 10 days, which was confirmed by lack of symptoms, signs and *M. genitalium* DNA at follow up visit four weeks after the treatment.

### Treatment failures with josamycin (n = 3)

Briefly, at the time of diagnosis two of the three patients had severe symptoms of urethritis and high number of PMNL/hpf, however, the remaining patient was asymptomatic with <5 PMNL/hpf. In the two symptomatic patients, the symptoms and signs initially resolved. However, one of them had relapsed symptoms and signs of severe urethritis 10–14 days after treatment, which was also the case for the initially asymptomatic patient. The *M. genitalium* strains in all three patients had mutations in the macrolide resistance region of 23S rRNA during treatment. Accordingly, the *M. genitalium* strains in two and one of those patients had an A2059G mutation (*Escherichia coli* numbering) and A2062G mutation, respectively, in the 23S rRNA gene (Table 3). The patients were subsequently successfully treated with moxifloxacin 400 mg daily, 10 days.

## Discussion

In the present study, it was shown that *M. genitalium* is a frequent cause of male urethritis in Moscow, Russia and that the *M. genitalium* eradication rate in 2006-2008 was 93.5% with the recommended first-line treatment in Russia, that is, josamycin 500 mg three times daily, 10 days [21]. These are the first data published regarding efficacy using josamycin for treatment of *M. genitalium* infections in the international (English language) literature. Accordingly, no evidence-based data supporting josamycin, compared to e.g. azithromycin, for treatment of *M. genitalium* infections and exceedingly limited *in vitro* data regarding josamycin susceptibility in *M. genitalium*, have been internationally published. To our best knowledge, the only *in vitro* antimicrobial susceptibility data were published in 1992 (only seven isolates were tested) and the activity was slightly lower compared to azithromycin [25]. In the present study, it was also shown that josamycin resistance resulting in treatment failures can rapidly be selected during treatment. The eradication rate of *M. genitalium* after the internationally widely used azithromycin 1 g therapy has substantially declined during the last decade [1,10,12-14,16]. Treatment with azithromycin 1 g was also recently associated with selection of macrolide resistant *M. genitalium* [14], and it was also hypothesized that treatment failures are frequently associated with a higher load of *M. genitalium* [17]. The results of the present study, instead examining the macrolide josamycin, are in full concordance with these recent findings. Accordingly, three failures to treat male *M. genitalium* urethritis with josamycin were verified. All of these patients had a high pre-treatment *M. genitalium* load (>6 geq/mL [log_10_]) and *M. genitalium* cells with mutations in the macrolide resistance-determining region V of 23S rRNA [1,10,13,14,18] were selected during treatment. Thus, despite that no 23S rRNA gene mutations were identified in the pre-treatment *M. genitalium* specimens the term selected resistance instead of induced resistance was used in the present study. Accordingly, it was considered that an undetected minority population of macrolide resistant *M. genitalium* cells might have been present already pre-treatment. These resistant cells, in the specimens with high *M. genitalium* load, could subsequently be selected during treatment with the bacteriostatic josamycin. The probability of this selection scenario was considered higher particularly because it is unknown how frequently josamycin might be able to directly induce 23S rRNA gene mutations, especially during treatment of *M. genitalium* with a very long generation time. The three identified treatment failures were further supported by the rebound in *M. genitalium* loads in all three patients and the recrudescence of symptoms in two of the three patients. Thus, the pre-treatment *M. genitalium* load might be an effective predictor of eradication efficacy with macrolides (and possibly additional antimicrobials) and selection of macrolide resistance due to mutations in the 23S rRNA gene. Unambiguously, additional data are crucial to support the recommendation of using josamycin regimen as first-line treatment in the Russian treatment guidelines for *M. genitalium* infections.

The most frequent 23S rRNA mutations identified in *M. genitalium* strains resistant to the 15-membered macrolide azithromycin have been A2058G and A2059G [1,13,14,17,18,30,31]. In the present study, it was for the first time shown that the A2059G mutation is associated also with *M. genitalium* resistance to 16-membered macrolides, e.g. josamycin. It has been earlier demonstrated that the A2059G mutation results in high-level resistance to 16-membered macrolides in the closely related species *M. pneumoniae* [32]. The A2062G mutation found in the remaining treatment failure has not been previously described in *M. genitalium*. However, *M. hominis* and *M. pneumoniae* cells with A2062G and A2062T mutations in the 23S rRNA gene have been previously selected *in vitro* using josamycin and shown to cause high-level josamycin resistance [33,34]. Interestingly, previous studies have suggested that mutations in the A2062 position of the 23S rRNA gene result in resistance to the 16-membered macrolides (e.g., josamycin), but not necessarily to the 15-membered macrolides (e.g., azithromycin), indicating a slightly different binding site [35-37]. In *M. pneumoniae*, the A2062G mutation, detected after *in vitro* exposure of josamycin, has also been shown to significantly increase the MICs of the streptogramin pristinamycin [34]. This is of grave concern because pristinamycin is highly active against *M. genitalium* infections and currently the only treatment option for infections resistant to both azithromycin and moxifloxacin [38]. Nevertheless, it remains unknown if an A2058G/A2059G mutation (resulting in azithromycin resistance) and an A2062G mutation (resulting in resistance to pristinamycin (and josamycin)) can co-exist in the 23S rRNA gene of *M. genitalium*, i.e. without severely decreasing the biological fitness and possibilities for transmission of the *M. genitalium* strain*.*

## Conclusions

In the present study, it was shown that *M. genitalium* is a frequent cause of male urethritis in Moscow, Russia. Furthermore, the first data on efficacy using josamycin for treatment of *M. genitalium* urethritis were also presented (93.5% eradication rate). All the three verified treatment failures had a high pre-treatment *M. genitalium* load and cells with mutations in the macrolide resistance region of 23S rRNA were easily selected during treatment. Because the present study examined material from December 2006 to January 2008 and the population selective pressure for resistance due to wide usage of macrolides has been very large and selected for relatively high-levels of azithromycin resistance in many countries during the recent decade, it is crucial to examine the josamycin treatment efficacy and *M. genitalium* load, including cut-off for possible treatment failure, as well as to screen for resistance to macrolides (and other antimicrobials) in a more recent material of *M. genitalium* positive clinical specimens (pre- and post-treatment) in Russia. This should ideally include both genetic screening for macrolide resistance mutations (previously described and novel), moxifloxacin resistance mutations and culture of *M. genitalium*, including MIC determination for macrolides (including azithromycin and josamycin), moxifloxacin and pristinamycin to confirm the phenotypic effects of the mutations on the antimicrobial resistance. No evidence-based data supporting josamycin, compared to e.g. azithromycin, for treatment of *M. genitalium* infections and exceedingly limited *in vitro* data regarding josamycin susceptibility in *M. genitalium*, have been internationally published [25]. Accordingly, additional *in vivo* and *in vitro* data are crucial to support the currently recommended josamycin regimen as first-line treatment in the Russian treatment guidelines for *M. genitalium* infections. It would be valuable to develop and implement international guidelines for management of *M. genitalium* infections and the results of the present study indicate that quantitative diagnostic PCRs determining also *M. genitalium* load and resistance mutations (for macrolides and ideally also moxifloxacin) should ideally be recommended for diagnosis of *M. genitalium* infections. Finally, novel options for effective treatment of *M. genitalium* infections are essential and dual antimicrobial therapy, which has been introduced for gonorrhoea, might need to be considered also for *M. genitalium* infections.
